# Clinicopathologic Evaluation of CD80, CD86, and PD-L1 Expressions with Immunohistochemical Methods in Malignant Melanoma Patients

**DOI:** 10.5146/tjpath.2023.01608

**Published:** 2024-01-22

**Authors:** Esra Cobankent Aytekin, Betul Unal, Cumhur Ibrahim Bassorgun, Ozlenen Ozkan

**Affiliations:** Department of Pathology, Konya Numune Hospital, Konya, Turkey; Department of Pathology, Akdeniz University, Faculty of Medicine, Antalya, Turkey; Department of Plastic and Reconstructive Surgery, Akdeniz University, Faculty of Medicine, Antalya, Turkey

**Keywords:** CD80, CD86, Immunopathology, Melanoma, PD-L1

## Abstract

***Objective: *** Diagnostic and prognostic biomarkers for malignant melanoma are crucial for treatment and for developing targeted therapies. Malignant melanoma is a highly immunogenic tumor, and its regression, treatment, and prognostic evaluation are directly related to escape from immune destruction. Therefore, we aimed to determine the expression levels of CD80, CD86, and PD -L1 in malignant melanoma tissue samples by immunohistochemistry and to investigate the possible relationship between these proteins and the clinicopathological features in this study.

***Material and Methods:*** Hematoxylin and eosin staining and immunohistochemical staining for CD80, CD86, and PD-L1 were evaluated for clinical data, survival, prognosis, tumor location, malignant melanoma subtypes, tumor size, and prognostic findings.

***Results: *** Higher survival rates were observed in patients with lower PD-L1 staining scores in the tumor. The 5-year survival was higher in patients with CD80-positive and CD86-positive biopsies. Mortality was lower in superficial spreading melanoma and Lentigo maligna melanoma types, whereas staining positivity of CD80 and CD86 was higher. Furthermore, a relationship between clinical stage and Breslow thickness (<2mm/≥2mm), tumor ulceration, lymph node metastasis, and CD80 and CD86 expression was also identified.

***Conclusion:*** Our findings suggest that PD-L1, CD80, and CD86 expression are essential in malignant melanoma and could be used as prognostic markers.

## INTRODUCTION

Malignant melanoma (MM) is a tumor with high immunogenicity due to tumor antigens (1). Because of this feature of MM, treatment protocols are centered on immunotherapeutic approaches, which include targets such as induction of anti-tumor immune responses, modulation of tumor-targeted immune reactions, and inhibition of immune control pathways (1).

Like many other tumors, tumor cells in MM inactivate the immune system through various escape mechanisms (2). The change in co-stimulatory receptors on dendritic cells is one of these escape mechanisms. The two-signal model proposes that activation of naive T cells requires both stimulation of T cell receptor (TCR) by major histocompatibility complex (MHC)-peptide molecules (signal 1) and co-stimulation via co-stimulatory receptors and their corresponding ligands on antigen-presenting cells (APCs) (signal 2). In this pathway, the tumor reduces (downregulates) the number of activating co-stimulatory receptors (CD28, CD40, OX40, CD137) or increases (upregulates) the number of inhibitory surface receptors (LAG-3, CTLA4, B7-H3, PD-1) in dendritic cells (1,3).

Programmed cell death receptor-1 (PD-1), a co-inhibitor, is expressed in T, B, and some myeloid cells. PD-L1 and PD-L2 are ligands expressed by various cells, including tumor cells, monocyte-derived myeloid dendritic cells, epithelial cells, and T and B cells (3–5). PD-L1/PD-1 interactions inhibit T cell growth and cytokine production. Furthermore, tumor cell PD-L1 can inhibit or cause apoptosis of tumor-specific T cells (4).

Immune T cells detect and respond to antigens presented by MHC on antigen-presenting cells and tumor cells. Co-activator signals are required for full activation of the T cell response. T-cell activity is inhibited when B7 of antigen-presenting cells (APC) binds to CTLA-4 of T-cells. B7 proteins are classified into two types: B7-1, also known as CD80, and B7-2, also known as CD86. CD28 and CTLA-4 (CD152) proteins can interact with B7-1 and B7-2 (6).

Recent research has revealed that CD80 and CD86 have both immunological (7,8) and non-immunological functions (9,10) and that CTLA-4 can act as an inhibitor independently of CD80 and CD86 (11). In addition, CD80 and CD86 are also involved in anti-tumor immunity (12–14).

Although studies have shown the expression of CD80 and CD86 in tumor cells, the related studies are limited, and their relationship with prognosis is unclear (2). As a result, the goal of this study was to look at the expression rates of CD80, CD86, and PD-L1 on paraffin sections from 80 malignant melanoma cases using immunohistochemistry, as well as to evaluate the possible correlation between these proteins and clinical features like the stage, prognosis, and survival, in order to see if these proteins can be used as prognostic markers and to shed light on new treatment modalities.

## MATERIAL and METHODS

Between 2005 and 2015, 80 patients diagnosed with MM were included in this study. The samples, of which 2 were incisional biopsies and 75 were excisional biopsies, were fixed in 10% buffered formalin, embedded in paraffin, cut into 4 mm thick sections, and stained with hematoxylin and eosin. A total of 3 consultation paraffin blocks were cut into 4 mm thick sections and stained with hematoxylin and eosin. In the presence of more than one block, we chose the block with the highest tumor ratio. In addition, demographic data such as age, gender, and clinical findings were retrieved from the files using the hospital automation system.

### Immunohistochemical Examination

Four-micrometer-thick sections were cut from each patient’s paraffin block. One of these sections was stained with hematoxylin and eosin (HE), and the others were immunohistochemically stained with antibodies against CD80 (Anti-CD80 antibody [2A2], 1/800, Abcam, UK), CD86 (Anti-CD86 antibody [EP1158Y], 1/800, Abcam, UK), and PD-L1 (Anti-PD-L1 antibody [ABM4E54], 1/1000, Abcam, UK). Two pathologists evaluated the stained sections. (CIB, ECA) We included a third pathologist in the study if the two pathologists could not reach a consensus about the diagnosis.

In this study, we developed a method of evaluating and scoring inspired by studies by Flörcken et al (2). Positive staining for CD80 and CD86 in tumor cells was characterized by cytoplasmic and membranous staining (2). PD-L1 was considered as positive when complete or partial linear membranous staining and nuclear staining were observed in tumor cells and tumor-infiltrated lymphocytes. Tonsillar tissue sections were positive controls for all PD-L1, CD80, and CD86 immunostainings (2,15,16).

We scored the staining intensity for CD80, CD86, and PD-L1. A score of 0 indicated no staining, while a score of +1 indicated weak staining. A score of +2 was for medium staining, and a score of +3 was given for intense staining. ≤10% positive staining of tumor cells was evaluated as 1 point, 10.1-50% positive staining as 2 points, and 50.1-100% positive staining as 3 points. The staining value was calculated by adding the staining percentage and intensity values. The patients were divided into groups based on their staining scores: those with a score of 0-4 and those with 5-6. Cases in the study were also classified as positive or negative for CD80 and CD86. The immunohistochemical staining results were evaluated based solely on the presence or absence of lymphocytic infiltration; however, no assessments were made regarding whether the infiltration was brisk or non-brisk.

### Statistical Evaluation

Descriptive findings were presented as number and percentage distributions for categorical variables, and mean±standard deviation for continuous variables.

The Pearson Chi-square test was used to compare categorical variables. For survival analysis, survival probabilities were first estimated with the Kaplan-Meier method and a log-rank test was performed to see if there was a difference between variable levels in terms of survival probabilities.

The University Institute of Health Sciences, Medical Statistics Consultancy Center used SPSS Package Program v20 to conduct the statistical analysis for the study (IBM, USA). Statistical significance was defined as p values less than 0.05.

## RESULTS

### Clinicopathological Characteristics

The median age of the patients was 57 years (range: 25-89 years). Among the study group, the death rate was 46.8% (n=22) in males, and 33.3% (n=11) in females, with no statistically significant difference between the two genders (p=0.256). Based on the World Health Organization’s classification of malignant melanoma, we identified 18 melanocytic tumors in intermittently sun-exposed skin (Superficial spreading melanoma), six melanocytic tumors in chronically sun-exposed skin (5 Lentigo maligna melanoma +1 desmoplastic melanoma), 20 melanoma arising at sun-shielded sites or without known etiological associations with UV radiation exposure (19 acral lentiginous melanoma and 1 balloon cell melanoma), and 36 nodular melanoma (17).

The patients in the study had a median tumor size of 1.78 cm in diameter (min: 0.2 cm, max: 7.6 cm), with 12.5% (n=10) having tumors smaller than or equal to 0.6 cm in diameter and 87.5% (n=70) having tumors larger than 0.6 cm in diameter. On the other hand, the patients in the study were divided into two groups based on reticular dermis invasion, Clark I-II, and Clark III-IV-V, with the Clark level I-II group accounting for 11.3 % of all patients (n=9). The Clark level III-IV-V group accounted for 88.7% (n=71). The mean Breslow thickness in all patients was 3.6.±3.24 mm: 37.5% (n=30) having a Breslow thickness of <2 mm, and 62.5% (n=50) having a Breslow thickness of ≥2 mm. Ulceration was found in 37.5 % of the patients (n=30). Regarding growth phases, 7.5 % of patients (n=6) showed only the radial growth phase, while 92.5 % (n=74) showed both the vertical/radial and vertical growth phases. Lymphocytic infiltration was found in 58.8% (n=47) of the patient samples examined. There was evidence of neurotropism in 23.8% of the cases (n=19). Regression was seen in 18.7% (n=15) of the patients, while lymph node metastasis was seen in 20% (n=16). 51.2 % (n=41) of the patients in the study were in Stages 1-2, while 48.8 % (n=39) were in Stages 3-4. The patients’ mean follow-up period was 57.72 ± 30.32 months (min: 0; max: 121 months). The mortality rate was 41.3% (n=33) during the follow-up period. Superficial spreading melanoma and lentigo maligna melanoma types, patients with Breslow thickness less than 2mm, the group without tumor ulceration, those with only radial growth phase, and clinical stage 1-2 all had lower mortality.

### Clinicopathological Correlations with Immunohistochemistry Results

#### Expression of PD-L1

**Figure 1:**
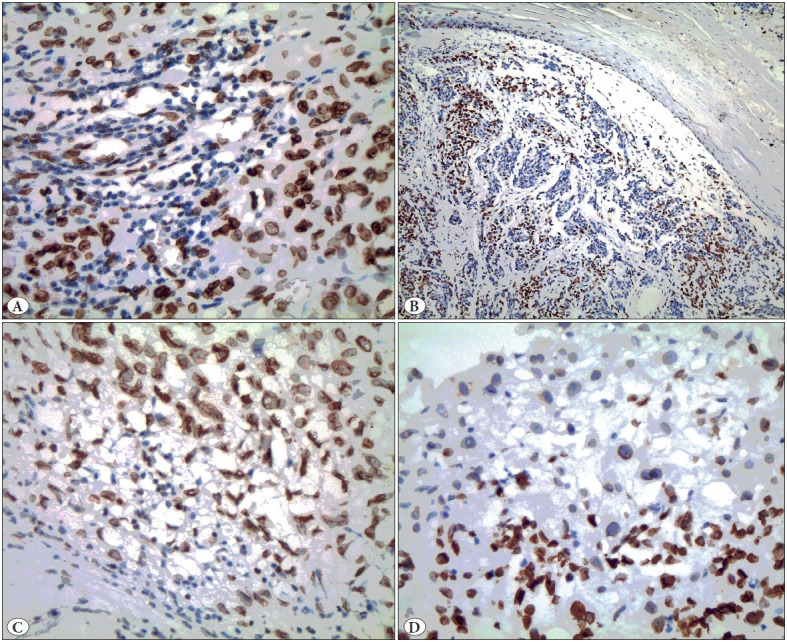
**A)** Strong membranous and nuclear immunohistochemical PD-L1 staining in tumor cells (PD-L1x400), **B)** Strong immunohistochemical PD-L1 staining in lymphocytes, negative staining in spindle tumor cells (x200), **C)** Membranous and nuclear immunohistochemical PD-L1 staining in tumor cells, negative staining in lymphocytes (PD-L1x400), **D)** Weak nuclear immunohistochemical PD-L1 staining in tumor cells, strong positive staining in lymphocytes (PD-L1x400).

As previously described, PD-L1 staining was assessed on tumor cells and tumor-infiltrating lymphocytes (2). Tumor cells had a low staining score (28/80) in 35% of the patients and a high staining score (52/80) in 65% of the patients, while tumor-infiltrating lymphocytes (TIL) had a low staining score (34/80) in 42.5% of the patients and a high staining score (46/80) in 57.5% of the patients. Figure 1 shows examples of low and high staining scores in tumors and TIL. PD-L1 expression and staining score were high in clinical stage 3-4 cases.

**Figure 2:**
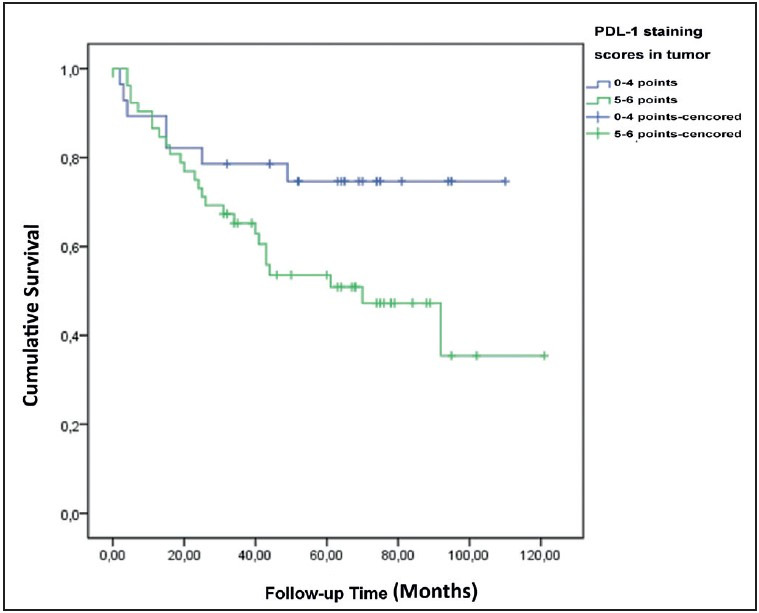
Survival plot according to PD-L1 staining scores in the tumor.

**Figure 3:**
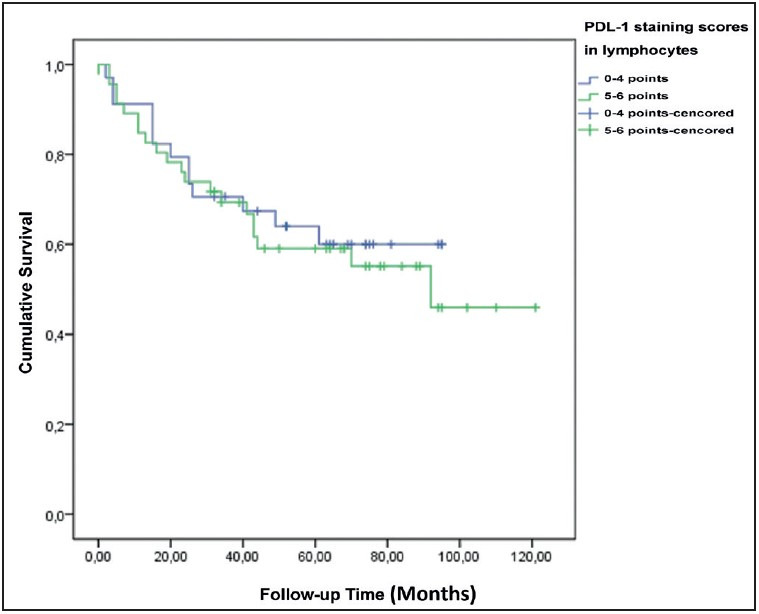
Survival plot according to PD-L1 staining scores in lymphocytes.

Patients with low PD-L1 staining in the tumor had a significantly higher 5-year survival (Figure 2). However, no significant relationship existed between the PD-L1 staining levels in the lymphocytes and the 5-year survival (Figure 3).

Table 1 summarizes the relationships between PD-L1 expression levels and the clinical profiles of the patients.

**Table 1:** The correlations between PD-L1 expression level and clinical patient profiles.

		**PD-L1 Staining Score in Tumor**					**PD-L1 Staining Score in Lymphocytes**				
		**0-4 points**		**5-6 points**		*p value	**0-4 points**		**5-6 points**		**p** **value**
		**Number**	**%**	**Number**	**%**		**Number**	**%**	**Number**	**%**	
Gender	Women	11	33.3	22	66.7	0.793	15	45.5	18	54.5	0.654
	Men	17	36.2	30	63.8		19	40.4	28	59.6	
Age (mean±S.D.)		56.71±18.94		57.88±14.37		0.445	56.26±17.55		58.36±14.9		0.709
Tumor size (mean±S.D.)		1.77±0.86		1.79±1.24		0.622	1.82±0.85		1.76±1.29		0.307
Clark Level	I and II	5	55.6	4	44.4	0.265	5	55.6	4	44.4	0.484
	III, IV and V	23	32.4	48	67.6		29	40.8	42	59.2	
Breslow thickness	Below 2mm	12	40	18	60	0.486	13	43.3	17	56.7	0.907
	Over 2mm	16	32	34	68		21	42	29	58	
Ulceration	No	19	38	31	62	0.468	19	38	31	62	0.293
	Yes	9	30	21	70		15	50	15	50	
Growth Phase	Radial	1	16.7	5	83.3	0.659	1	16.7	5	83.3	0.233
	Vertical or Radial+vertical	27	36.5	47	63.5		33	44.6	41	55.4	
Lymphocytic Infiltration	No	5	23.8	16	76.2	0.211	8	38.1	13	61.9	0.634
	Yes	23	39	36	61		26	44.1	33	55.9	
Clinical Stage	Stages 1-2	21	51.2	20	48.8	**0.002**	21	51.2	20	48.8	0.106
	Stages 3-4	7	17.9	32	82.1		13	33.3	26	66.7	
Lymph node metastasis	No	22	34.4	42	65.6	0.815	26	40.6	38	59.4	0.497
	Yes	6	37.5	10	62.5		8	50	8	50	
Neurotropism	No	22	36.1	39	63.9	0.72	26	42.6	35	57.4	0.968
	Yes	6	31.6	13	68.4		8	42.1	11	57.9	
Regression	No	22	33.8	43	66.2	0.652	26	40	39	60	0.346
	Yes	6	40	9	60		8	53.3	7	46.7	

*Pearson-chi square test

#### Expression of CD80

**Figure 4:**
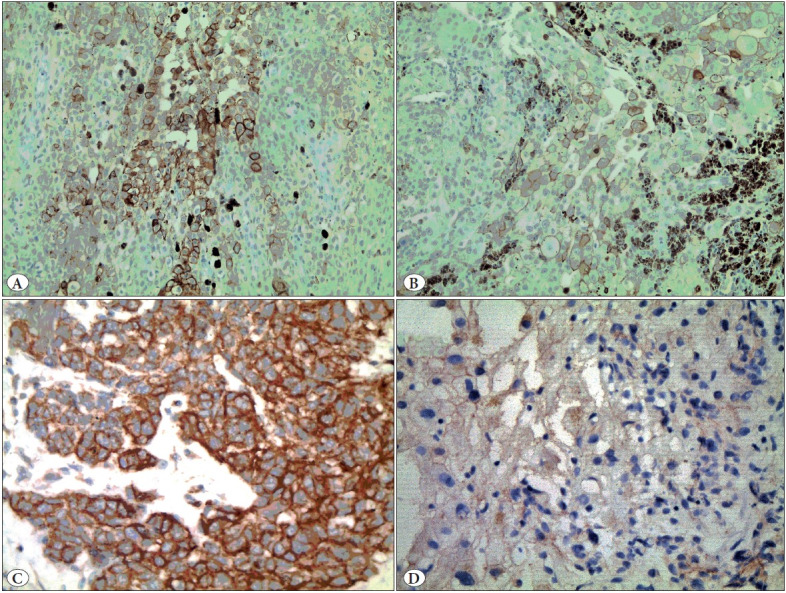
**A)** Strong membranous immunohistochemical CD80 staining in tumor cells (CD80 x200), **B)** Weak membranous immunohistochemical CD80 staining in tumor cells, (CD80 x200), **C)** Strong membranous immunohistochemical CD86 staining in tumor cells (CD86 x400), **D)** Weak membranous immunohistochemical CD86 staining in tumor cells (CD86 x400).

CD80 expression (staining intensity) was detected in 45% (36/80) of the patients (Table 2). CD80 staining scores (Figure 4) ranged from 0 to 4 in 79% of the patients (63/80) and from 5 to 6 in 21% of the patients (17/80). Table 3 shows the correlations between CD80 expression levels and the clinical patient profiles.

**Table 2:** CD80 and CD86 expressions.

		**CD86**		
		**Positive (n)**	**Negative (n)**	**Total (n)**
**CD80**	**Positive**	35	1	36
	**Negative**	2	42	44
	**Total**	37	43	80

**Table 3:** The correlations between CD80 expression level and clinical patient profiles.

		**CD80 Expression**					**CD80 Staining Score**				
		**No**		**Yes**		*p value	**0 – 4 points**		**5-6 points**		**p** **value**
		**Number**	**%**	**Number**	**%**		**Number**	**%**	**Number**	**%**	
Gender	Women	17	51.5	16	48.5	0.6	25	75.8	8	24.2	0.584
	Men	27	57.4	20	42.6		38	80.9	9	19.1	
Age (mean±S.D.)		56.69±15.26		58.55±17.03		0.588	57.39±15.18		57.6±19.29		0.934
Tumor size (mean±S.D.)		1.9±1.32		1.65±0.80		0.568	1.83±1.16		1.63±0.95		0.545
Clark Level	I and II	23	33.3	6	66.7	0.286	6	66.7	3	33.3	0.392
	III, IV and V	3	57.7	30	42.3		57	80.3	14	19.7	
Breslow thickness	Below 2mm	41	33.3	20	66.7	**0.003**	19	63.3	11	36.7	**0.009**
	Over 2mm	10	68	16	32		44	88	6	12	
Ulceration	No	34	40	30	60	**0.0001**	36	72	14	28	0.089
	Yes	20	80	6	20		27	90	3	10	
Growth Phase	Radial	24	16.7	5	83.3	0.085	5	83.3	1	16.7	1
	Vertical or Radial+vertical	1	58.1	31	41.9		58	78.4	16	21.6	
Lymphocytic Infiltration	No	43	52.4	10	47.6	0.779	14	66.7	7	33.3	0,13
	Yes	11	55.9	26	44.1		49	83.1	10	16.9	
Clinical Stage	Stages 1-2	33	26.8	30	73.2	**<0.001**	28	68.3	13	31.7	**0.028**
	Stages 3-4	11	84.6	6	15.4		35	89.7	4	10.3	
Lymph node metastasis	No	31	48.4	33	51.6	**0.024**	47	73.4	17	26.6	**0.018**
	Yes	13	81.2	3	18.8		16	100	0		
Neurotropism	No	32	52.5	29	47.5	0.413	49	80.3	12	19.7	0.534
	Yes	12	63.2	7	36.8		14	73.7	5	26.3	
Regression	No	32	49.2	33	50.8	**0.030**	51	78.5	14	21.5	1
	Yes	12	80	3	20		12	80	3	20	

*Pearson-chi square test

When CD80 expression was examined, it was significantly higher in the superficial spreading melanoma and lentigo maligna melanoma subtypes than in other subtypes.

CD80 staining scores were higher in superficial spreading melanoma and lentigo maligna melanoma subtypes when evaluating CD80 staining scores.

CD80 expression (staining intensity) and staining scores were higher in patients with Breslow thickness below 2 mm.

The positivity for CD80 expression was higher in patients who did not have tumor ulceration.

**Figure 5:**
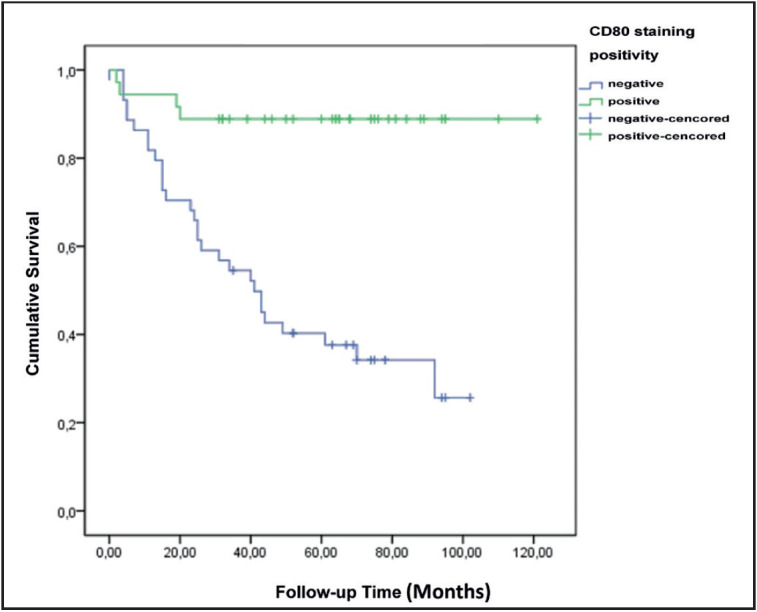
Survival plot according to CD80 staining positivity.

**Figure 6:**
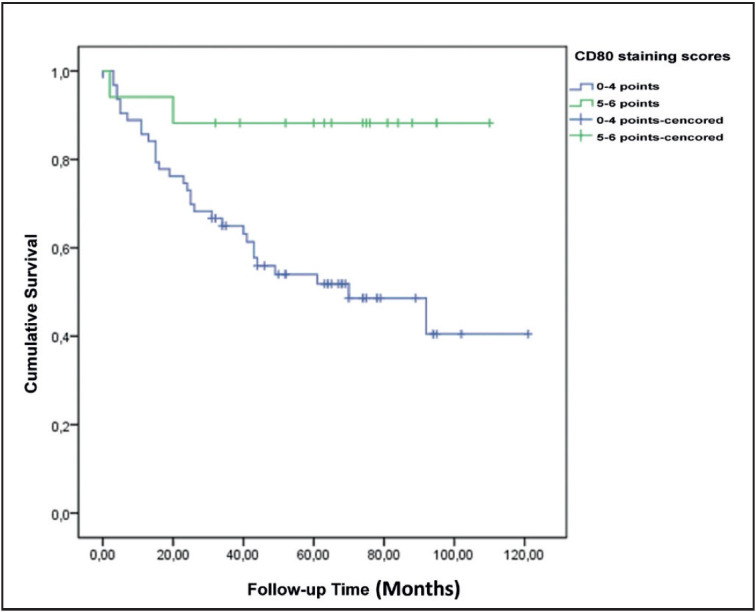
Survival plot according to CD80 staining scores.

CD80 expression was found to be higher in cases of regression. CD80 expression was higher in patients who did not have lymph node metastasis. CD80 expression and staining scores were higher in clinical stage 1-2 cases. Cases with positive CD80 expression had a significantly higher 5-year survival rate (Figure 5 and Figure 6).

#### CD86 expression

CD86 expression was found in 46.25% of the cases (37/80) (Table 2). CD86 staining (Figure 4) scores ranged from 0 to 4 points (64/80) in 80% of the patients and from 5 to 6 points in 20% (16/80).

Table 4 shows the correlations between CD86 expression levels and the clinical profiles of the patients. When CD86 expression was examined, it was significantly higher in superficial spreading melanoma and lentigo maligna melanoma than in other subtypes.

**Table 4:** The correlations between CD86 expression level and clinical patient profiles.

		**CD86 Expression**					**CD86 Staining Score**				
		**No**		**Yes**		*p **value**	**0 – 4 points**		**5-6 points**		**p** **value**
		**Number**	**%**	**Number**	**%**		**Number**	**%**	**Number**	**%**	
Gender	Women	18	54.5	15	45.5	0.985	26	78.8	7	21.2	0.82
	Men	25	53.2	22	46.8		38	80.9	9	19.1	
Age (mean±S.D.)		56.44±14.60		58.67±17.63		0.537	55.81±15.64		64.12±16.22		0.063
Tumor size (mean±S.D.)		1.86±1.33		1.69±0.82		0.911	1.77±1.18		1.86±0.86		0.43
Clark Level	I and II	4	44.4	5	55.6	0.726	5	55.6	4	44.4	0.073
	III, IV and V	39	54.9	32	45.1		59	83.1	12	16.9	
Breslow thickness	Below 2mm	11	36.7	19	63.3	**0.018**	20	66.7	10	33.3	**0.021**
	Over 2mm	32	64	18	36		44	88	6	12	
Ulceration	No	21	42	29	58	**0.007**	39	78	11	22	0.564
	Yes	22	73.3	8	26.7		25	83.3	5	16.7	
Growth Phase	Radial	1	16.7	5	83.3	0.058	5	83.3	1	16.7	1
	Vertical or Radial+vertical	42	56.8	32	43.2		59	79.7	15	20.3	
Lymphocytic Infiltration	No	12	57.1	9	42.9	0.717	17	81	4	19	1
	Yes	31	52.5	28	47.5		47	79.7	12	20.3	
Clinical Stage	Stages 1-2	12	29.3	29	70.7	**0.0001**	29	70.7	12	29.3	**0.034**
	Stages 3-4	31	79.5	8	20.5		35	89.7	4	10.3	
Lymph node metastasis	No	30	46.9	34	53.1	**0.014**	48	75	16	25	**0.032**
	Yes	13	81.2	3	18.8		16	100	0	0	
Neurotropism	No	32	52.5	29	47.5	0.678	50	82	11	18	0.514
	Yes	11	57.9	8	42.1		14	73.7	5	26.3	
Regression	No	18	54.5	15	45.5	0.15	26	78.8	7	21.2	1
	Yes	25	53.2	22	46.8		38	80.9	9	19.1	

*Pearson-chi square test

CD86 expression and a higher CD86 staining intensity score were found in patients with a Breslow thickness of less than 2mm.

CD86 expression was significantly higher in tumors that did not have ulceration. The expression of CD86 was higher in patients who did not have lymph node metastasis.

CD86 expression and CD86 staining scores were significantly higher in pathological Stages 1-2 cases.

**Figure 7:**
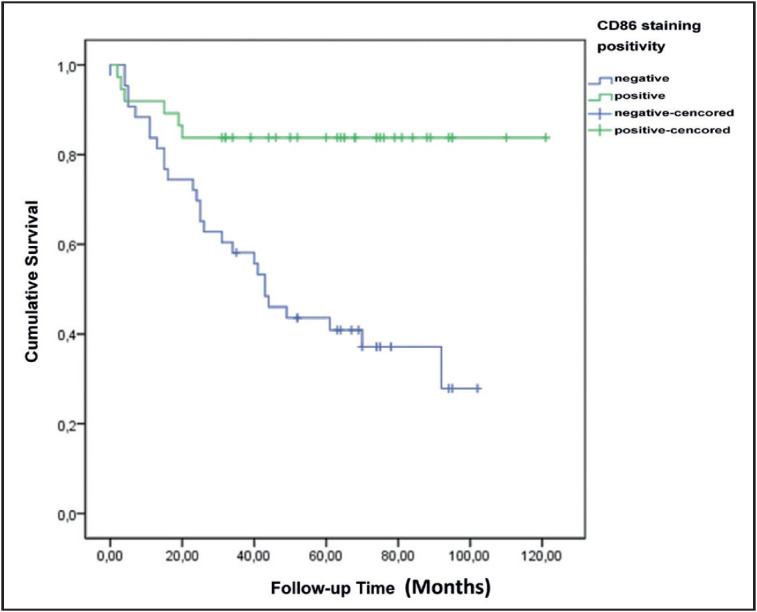
Survival plot according to CD86 staining positivity.

**Figure 8:**
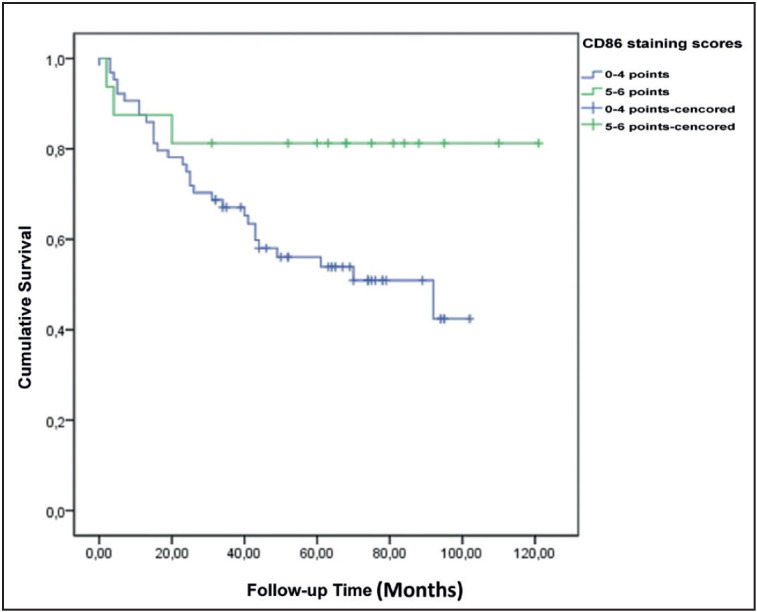
Survival plot according to CD86 staining scores.

While patients with positive CD86 expression had a significantly higher 5-year survival rate, there was no correlation between staining score and survival (Figure 7 Figure 8).

The simultaneous presence of CD80 and CD86 positivity or negativity was found to be significantly higher (there was concurrent CD86 expression in cases with CD80 expression or absent CD86 expression in cases without CD80 expression).

## DISCUSSION

Malignant melanoma is a highly immunogenic tumor and its progression, treatment, and prognosis are directly related to this feature (1). Antagonistic monoclonal antibodies against CTLA-4 and PD-1 have been introduced for immune checkpoint blockade. These agents’ anti-tumor activities have been demonstrated in Phase I, II, and III studies, with CTLA-4 and PD-L1 standing out as the primary molecules targeted by immunotherapy modalities results (2).

Although most studies on the inhibitory or stimulatory effects of signal-2 in the formation of the immune response focus on the two molecules mentioned above, the results of the CD80/CD86-CD28 interaction on immune modulation are also known.

Our retrospective clinical study investigated the association between CD80, CD86, and PD-L1 expression, tumor characteristics, prognostic factors, and survival in cutaneous malignant melanoma.

In the patients enrolled in our study, no significant relationship was found between the tumor size and Clark level, as well as the CD80, CD86, and PD-L1 expression levels. However, it has been suggested that the immune response to the tumor effects the vertical growth phase rather than the radial growth phase because the mortality rate is high in patients with a Breslow thickness of 2 mm or more, and the expression of CD80 and CD86 is significantly lower in these cases.

There was no statistically significant relationship between PD-L1 staining intensity and Breslow thickness. However, the Breslow thickness was significantly higher in patients with strong PD-L1 expression compared to those with weak expression in Hino and colleagues’ study (15). In addition, PD-L1 expression was also associated with vertical growth pattern, Clark level status, and lymph node metastasis but not with age, sex, or histologic subtype in the same study (15).

Hino and colleagues found no link between ulceration and PD-L1 expression, and Massi and colleagues found ulceration to be a poor predictor of survival. Their findings were consistent with ours (15,16).

Our study discovered no link between lymphocytic infiltration and mortality, nor between CD80, CD86, and PD-L1 expression and lymphocytic infiltration. Consistent with our findings, Massi and colleagues reported no relationship between lymphocytic infiltration and PD-L1 expression. In addition, no significant relationship between lymphocytic infiltration and survival was found in the reports of Gadiot and colleagues (16,18).

PD-L1 expression has been identified in melanoma, ovarian tumors, lung tumors, renal cell tumors, urothelial tumors, squamous cell carcinomas of the head and neck, esophageal tumors, cervical tumors, breast tumors, pancreatic tumors, stomach tumors, Wilms tumors, and glioblastoma, as well as infiltrating lymphocytes (15,19–30). The literature has no consensus regarding the relationship between membranous expression and survival. While several studies have found PD-L1 expression in tumors to be statistically significantly associated with poor prognosis and survival (16,19,31–36), some studies show the opposite (18,24,29).

Similar to previous research suggesting that PD-L1 in tumor cells can be used as a poor prognostic marker based on the presence or absence of staining, we found that strong staining of PD-L1 in tumor tissue indicated poor prognosis and survival. However, this was not found with PD-L1 expression in TIL. There is little evidence in the literature to support the use of CD80 and CD86 immunohistochemistry in tumor immunity and prognosis. While most rodent tumors have been reported to lack tumor expression of CD80 and CD86 (37,38), molecular, immunohistochemical, and flow cytometry studies have revealed the presence of CD80 and CD86 in tumor cells in some human tumor cells (39–41).

PD-L1 expression in tumor cells and lymphocytes was associated with a poor prognosis in a study evaluating the expression of CD80, CD86, and PD-L1 in both the tumor and lymphocytes in renal cell tumor cases in the literature, whereas CD80 and CD86 expressions were not correlated with the prognosis (2). In support of these reports, publications indicate that CD80 and CD86 expression in melanoma patients does not effect the prognosis (42).

CD80 and CD86 expression on the cell surface was significantly positive in cases with Breslow thickness less than 2mm, no ulceration on the tumor surface, clinical stage I-II, and no lymph node metastasis in our study. Furthermore, CD80 and CD86 positivity was found to indicate a favorable prognosis.

When analyzed, only CD80 positivity and the presence of regression had a significant relationship. The failure to see any significant relationship for CD86 positivity is likely due to a small sample size.

Histologic subtype analysis revealed that CD80 and CD86 expression was significantly higher in superficial spreading melanoma and lentigo maligna melanoma.

Based on our findings, we recommend using PD-L1, expressed in tumor tissue, as a prognostic marker in cases of malignant melanoma, with a high PD-L1 staining score in the tumor indicating a poor prognosis and determining the indications for the patient’s clinical management and immunotherapy. CD80 and CD86 expressions and high staining scores were statistically significant predictors of a good prognosis in our study’s survival analysis. As a result, CD80 and CD86 immunohistochemical markers predict the prognosis of malignant melanoma cases**.**

## Conflict of Interest

No conflict of interest.

## Funding

Akdeniz University Scientific Research Projects Unit (Project number: TTU-2017-954).
